# Elaboration, Characterization and Thermal Decomposition Kinetics of New Nanoenergetic Composite Based on Hydrazine 3-Nitro-1,2,4-triazol-5-one and Nanostructured Cellulose Nitrate

**DOI:** 10.3390/molecules27206945

**Published:** 2022-10-17

**Authors:** Ahmed Fouzi Tarchoun, Djalal Trache, Amir Abdelaziz, Abdelatif Harrat, Walid Oussama Boukecha, Mohamed Abderrahim Hamouche, Hani Boukeciat, Mohammed Dourari

**Affiliations:** 1 Energetic Propulsion Laboratory, Teaching and Research Unit of Energetic Processes, Ecole Militaire Polytechnique, BP 17, Algiers 16046, Algeria; 2 Energetic Materials Laboratory, Teaching and Research Unit of Energetic Processes, Ecole Militaire Polytechnique, BP 17, Bordj El-Bahri, Algiers 16046, Algeria

**Keywords:** nanostructured nitrocellulose, hydrazine 3-nitro-1,2,4-triazol-5-one, nanoenergetic composite, compatibility, thermal kinetics

## Abstract

This research aims to develop new high-energy dense ordinary- and nano-energetic composites based on hydrazine 3-nitro-1,2,4-triazol-5-one (HNTO) and nitrated cellulose and nanostructured nitrocellulose (NC and NMCC). The elaborated energetic formulations (HNTO/NC and HNTO/NMCC) were fully characterized in terms of their chemical compatibility, morphology, thermal stability, and energetic performance. The experimental findings implied that the designed HNTO/NC and HNTO/NMCC formulations have good compatibilities with attractive characteristics such as density greater than 1.780 g/cm^3^ and impact sensitivity around 6 J. Furthermore, theoretical performance calculations (EXPLO5 V6.04) displayed that the optimal composition of the as-prepared energetic composites yielded excellent specific impulses and detonation velocities, which increased from 205.7 s and 7908 m/s for HNTO/NC to 209.6 s and 8064 m/s for HNTO/NMCC. Moreover, deep insight on the multi-step kinetic behaviors of the as-prepared formulations was provided based on the measured DSC data combined with isoconversional kinetic methods. It is revealed that both energetic composites undergo three consecutive exothermic events with satisfactory activation energies in the range of 139–166 kJ/mol for HNTO/NC and 119–134 kJ/mol for HNTO/NMCC. Overall, this research displayed that the new developed nanoenergetic composite based on nitrated cellulose nanostructure could serve as a promising candidate for practical applications in solid rocket propellants and composite explosives.

## 1. Introduction

In the last few decades, the development of new insensitive and high-energy dense composites has addressed a series of challenges in the field of materials chemistry and advanced applications (e.g., high performance solid propellants and insensitive composite explosives) [1,2]. For instance, the most newly developed energetic materials, are still not able to completely replace currently used ones in the military systems due to several problems including chemical incompatibility, low thermal stability, worse sensitivity, as well as high cost, which impede their further use in military systems [3,4]. As a common branch of energetic materials, nitrocellulose (NC)-based formulations play a prominent role in a broad range of industrial (e.g., membranes and cosmetic products) and defense (e.g., smokeless gun powders and rocket propellants) areas owing to their easily tunable and tailorable characteristics such as excellent mechanical properties, compatibility with several additives, flammability, and explosiveness [5]. However, long-term insight with traditional NC-based energetic composites demonstrates some of its limits, including high impact sensitivity, low density, poor thermal stability, and inappropriate combustion performance, which restricted their practical application in advanced munitions [6,7]. For this purpose, the energetic materials community has driven investigations toward the structural downscaling technology of NC to design promising nanostructured nitrocellulose, known as nitrated microcrystalline cellulose (NMCC), offering new opportunities to develop innovative energetic mixtures with advanced functionalities. According to literature reports, this emergent subclass of nitrate esters nanostructured cellulose derivative displayed improved nitrogen content, density, crystallinity, thermal reactivity, and energetic performance compared to the traditional NC, which renders it a highly desirable alternative candidate for potential employment in modern energetic formulations [8,9].

In terms of solid propellants, the most important goal is to achieve higher energy release with controlled processes [2,10]. Therefore, the energetic performance, compatibility, thermal security, and vulnerability of rocket propellants, gun propellants, and gas generators containing various novel ingredients are the major research directions in this field [11,12]. As one of the key components, high-energy fillers demonstrate great potential for multipurpose application, especially in the development of high-performance propellants. In particular, nitrogen-rich compounds are widely evaluated for solid propellants and explosives formulations to meet the multiple requirements of high density, good thermal stability, insensitivity, and high-energy performance of munitions. In the case of NC-based propellants, nitroglycerine (NG) is considered the oldest and the most commonly used energetic component, whereas, its substitution has been in high demand due to its migratory issues and reduced stability [13,14]. In order to overcome these challenges and eliminate dependence on NG, numerous types of emergent high-energy dense additives comprising nitramines, energetic ionic liquids, green oxidizers, and energetic co-crystals have been tested for NC-based systems [15,16,17]. For instance, it has been demonstrated that the incorporation of nitramine explosives into the NC matrix greatly improves the detonation properties and specific impulse of munitions [18]. For these reasons, many kinds of NC matrix-based energetic composites have been manufactured and fully investigated, including NC/RDX (cyclotrimethylenetrinitramine) [19], NC/HMX (cyclotetramethylenetetranitramine) [20], NC/CL-20 (Hexanitrohexaazaisowurtzitan) [21], and NC/GAP (glycidyl azide polymer) [22], for eventual application in modern propellant and explosive systems. Nevertheless, a significant concern with nitrate esters cellulosic formulations having a high nitramines concentration, which are the significantly high sensitivity, the reduced interactions between the NC and energetic filler, and the elevated flame temperature that cause gun wear and thus reduce the useful life of the gun barrel. Therefore, the search for new NC mixtures with advanced capabilities and enhanced features is a crucial driving force to substantially alleviate many of the aforementioned issues.

Recently, a great deal of interest has focused on nitrogen-rich heterocyclic salts owing to their desirable properties such as low sensitivities, high-energy nature, excellent stability, and good compatibility with other energetic compounds [23]. In this context, hydrazine 3-nitro-1,2,4-triazol-5-one (HNTO), obtained by hydrazine hydrate modification of NTO, is an energetic salt that has been recently developed and acquired very attractive features such as high density (ρ = 1.820 g/cm^3^) and nitrogen content (*Nc* = 51.83%), good thermal stability, moderate impact sensitivity (7 J), and great energy content [24,25]. Moreover, its low acidity compared to NTO and its relatively acceptable production cost (≈85 €/100 g based on the market prices of the starting materials) with its low environmental impact indicated its high potential to be utilized in propellant and explosive formulations. Therefore, these outstanding features of HNTO have motivated us to scrutinize its ability to be employed with nitrated cellulosic polymers and examine the characteristics of the resulting composites. In addition, an investigation of the thermokinetic behaviors of the above-mentioned energetic composites would be very helpful to accurately evaluate their thermal stability and decomposition pathways, as well as estimate the effect of their thermal run-aways on the potential hazard during their preparation, processing, storage, and real-world applications.

The present study aims to elaborate on new ordinary- and nano-energetic composites consisting of nitrogen-rich heterocyclic salt and nitrocellulose and nanostructured nitrocellulose (HNTO/NC and HNTO/NMCC), and deeply investigate their chemical compatibility, morphological structure, and thermal behavior. In addition, their thermokinetic parameters were predicted for the first time based on non-isothermal DSC data and using three isoconversional integral methods to accurately judge their safety performance and offer guidelines for their potential employed in solid rocket propellants and composite explosives and solid propellants. The obtained results were compared to those of other nano-energetic formulations found in the literature as well.

## 2. Results and Discussion

### 2.1. DSC-Based Compatibility

**Figure 1:**
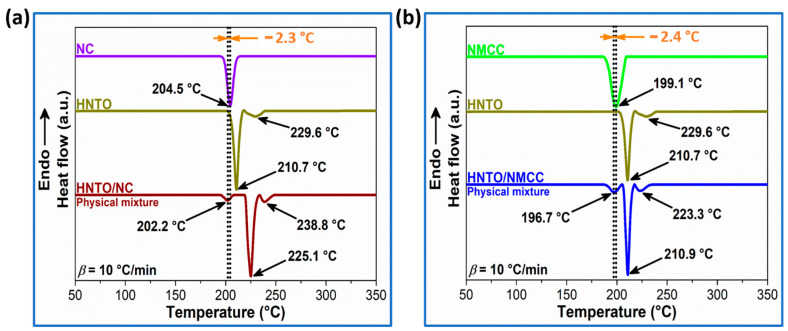
DSC thermograms at a heating rate of 10 °C/min of (**a**) HNTO/NC physical mixture and its single components; (**b**) HNTO/NMCC physical mixture and its single components.

The heat flow curves of the pure compounds and their physical mixtures are shown in Figure 1. As observed from the plotted DSC thermograms plotted, NC and NMCC polymers display only one exothermic process that occurred at maximum peak temperatures of 204.5 °C and 199.1 °C, respectively, which is assigned to the well-known thermolysis pathway of nitrated cellulosic chains [9,26]. Pure HNTO, however, undergoes two consecutive exothermic events within the range of 205–220 °C and 220–235 °C, similar to what is commonly reported in previous works [25,27]. For the physical mixtures, there were still three independent exothermic peaks, corresponding to the thermal decomposition of single nitrated cellulosic binders and HNTO, suggesting that there is no chemical reaction between components of the mixture. It is noteworthy that the maximum exothermic peak temperature of nitrated cellulosic polymers is lower than that of HNTO sample, so energetic NC and NMCC matrices are selected as the single compound in the mixture systems.

According to Figure 1, it is obvious that the thermal decomposition of NC and NMCC is shifted to a lower temperature in the HNTO/NC and HNTO/NMCC mixtures, respectively, indicating that the presence of HNTO has accelerated the thermolysis process of cellulosic nitrates. The maximum exothermic peak temperature difference between NC and HNTO/NC, NMCC and HNTO/NMCC is found to be 2.3 °C and 2.4 °C, respectively, showing that NC and NMCC are both compatible with HNTO according to the STANAG 4147 criterion [28]. These findings confirm the potential use of HNTO in nitrocellulose-based composite explosives and rocket propellants.

### 2.2. Determination of the Optimal Composition of the Energetic Formulations

To design high energetic and efficient formulations for potential applications in rocket propellant systems and composite explosives, one should select ingredients fractions that provide optimum values of *I_SP_* and *D_C-J_*. Therefore, EXPLO5 (V6.04) software (OZM Research, Version 6.04, Czech Republic, European Union), as a powerful thermochemical tool, is employed to select the best formulations based on HNTO/NC and HNTO/NMCC composites. Figure 8b shows the evolution of the computed *I_SP_* and *D_C-J_* of each composite as a function of HNTO content. It can be revealed that the increase of the HNTO content leads to a noticeable enhancement of the detonation velocity, which is attributed to the higher heat of formation and explosive nature of HNTO. However, the decreased trend of the specific impulse while increasing the HNTO fraction could be probably caused by its negative oxygen balance (−38%), which reduces the flame temperature of the composite that is directly proportional to the *I_SP_* [29,30]. Therefore, the intersection point between the *I_SP_* and *D_C-J_* curves is taken as the optimum HNTO mass fraction. A satisfactory specific impulse (*I_SP_* > 205 s) and relatively high detonation velocity (*D_C-J_* > 7900 m/s) are obtained for the optimal composition (60:40, wt.%) of HNTO/NC and HNTO/NMCC formulations. Another important finding is that HNTO/NMCC composite produces higher *I_SP_* and *D_C-J_* than HNTO/NC, highlighting the potential advantage of using emergent nanostructured cellulose nitrate instead of traditional cellulose nitrate to promote the energetic performance of the developed energetic cellulose-rich formulations. These results demonstrate the relationship between the morphology of NMCC and its physicochemical properties (nitrogen content and density) in one hand, and on the other hand the proportional effect of these parameters on the energetic performance.

On the other hand, it is interesting to point out that the optimal *D_C-J_* of the designed composites is found to be better than that of some common explosives, including TNT (6900 m/s) [31], NG (7823 m/s) [32], HNS (7612 m/s) [33], and other aluminized- and ammonium nitrate-bonded explosives reported by Suceska et al. [34]. With regard to the optimal specific impulse, it is found that the elaborated HNTO/NC and HNTO/NMCC composites deliver moderate *I_SP_*, which is comparable or slightly lower than some currently used homogenous and modified double base rocket propellants (220 s) [35,36], and other reported NC-based composites such as NC/GAP (205 s) [18], and NC/HMX (231 s) [20]. It is worth noting that the *I_SP_* of our designed composites can be significantly improved by the small addition of metal hybrids, which can offer extra oxidizing species that contribute to the total oxidation of the available fuel species, thus increasing the resulting ratio of combustion temperature to molar mass as demonstrated in our recent papers [30,37]. In light of these results, it can be inferred that the newly designed ordinary- and nanostructured-energetic composites (HNTO/NC and HNTO/NMCC) can be considered potential candidates for the next generation of energetic formulations.

### 2.3. Morphological and Structural Characterizations

The micro-scale morphology and the structure of the developed energetic HNTO/NC and HNTO/NMCC composites were assessed using SEM and FTIR, respectively.

**Figure 2:**
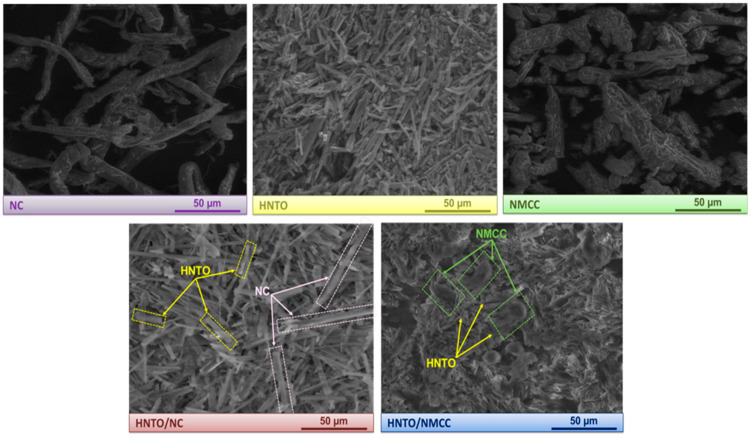
SEM micrographs of the elaborated energetic composites and their raw compounds.

As can be observed from the SEM micrographs depicted in Figure 2, NC displays long individualized filaments with a rough surface; NMCC shows irregular microstructure with rod-shaped aggregates, while HNTO presents a smooth irregular elongated rod-like shape structure. Regarding the morphological features of the prepared energetic composites, it is clear from Figure 2 that the microstructure of HNTO/NC is different from that of HNTO/NMCC. A well-defined structure of pure HNTO and NC can be clearly noticed for the double base HNTO/NC film, which indicates the strong intramolecular interactions between the rod-shaped HNTO and NC fibers. Meanwhile, the HNTO/NMCC composite exhibits an embedded rod-like microcrystals network with some spherical aggregates, demonstrating that the use of nanostructured NMCC matrix instead of traditional NC leads to more homogenous dispersion of HNTO particles to form a dense matrix. Such effective dispersion is broadly attributed to the enhanced interfacial contact between the different ingredients, which is expected to promote the thermal decomposition performance of the composite, as will be proved later in the next sections.

To further corroborate the above findings, the density of the elaborated energetic composites, which is a crucial parameter that may influence their energy performance, was experimentally determined using an electronic densimeter, and the obtained values are given in Table 1. The foremost finding is that the density of HNTO/NMCC (1.793 ± 0.002 g/cm^3^) is greater than that of the baseline HNTO/NC (1.781 ± 0.002 g/cm^3^), which is even superior to those of raw NC (1.671 ± 0.004 g/cm^3^) and NMCC (1.694 ± 0.004 g/cm^3^). These outcomes are in good accordance with those obtained by SEM and provide further evidence for the potential advantages of utilizing NMCC to develop promising high-density nanostructured formulations for futuristic energetic applications. Furthermore, it is found that the measured densities of HNTO/NC and HNTO/NMCC are quite similar to their theoretical values with a percentage gap (Δρ) lower than 2%, which reveals that the open porosity of the as-prepared formulations is negligible, hence confirming their homogeneity with no bubble-contamination production during the mixing process [37,38]. Besides that, it is interesting to point out that the designed composites based on hydrazine 3-nitro-1,2,4-triazol-5-one and nitrated cellulosic polymers have better densities than the commonly employed double base rocket propellants (1.55–1.66 g/cm^3^) [36,39], which are comparable or slightly higher than those of some reported composite propellants and explosives [40,41,42].

**Figure 3:**
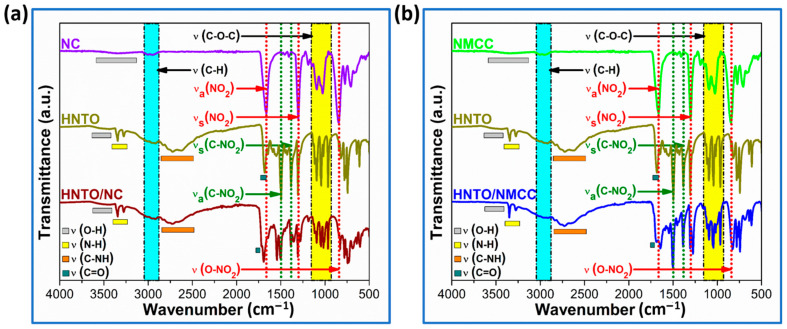
FTIR spectra of (**a**) HNTO/NC composite and its single components; (**b**) HNTO/NMCC composite and its single components.

On the other hand, the molecular structure of the new developed HNTO/NC and HNTO/NMCC composites and their raw compounds has also been identified by FTIR measurements with the aim of revealing the eventual changes of their structures and the appearance or disappearance of bonds by the effect of their reciprocal interactions, and the recorded spectra are presented in Figure 3. As can be revealed, both composites show the characteristic vibrational peaks of nitrated cellulosic chains, which are O-H stretching at 3500–3470 cm^−1^, C-H stretching at 2900 cm^−1^, and the absorption bands of NO_2_ and O-NO_2_ groups in the fingerprint region from 1800 cm^−1^ till 500 cm^−1^. Furthermore, the typical functional groups of HNTO are also identified in the spectra of the elaborated composites at representative bands of 3350–3280 cm^−1^ for primary N-H of hydrazine group, 2730 cm^−1^ for N-H in triazole ring, 1690 cm^−1^ for C=O, 1509 cm^−1^ and 1320 cm^−1^ for asymmetric and symmetric C-NO_2_, respectively [16,43]. Besides that, the slight red-shift of some chemical bands associated with HNTO salt is attributed to the interaction of electron-withdrawing nitrate esters linked to cellulose backbone with nitro and carbonyl groups of triazole heterocycle, which affect the electron density of atoms [16,25]. Similarly, the significant drop in the intensity of the OH band suggests the presence of hydrogen bonds between hydrazine moiety of HNTO molecule and nitrated cellulosic chains. These findings demonstrate that the main molecular structures of HNTO, NC, and NMCC were maintained during the fabrication process.

### 2.4. Assessment of the Thermal Behavior

**Figure 4:**
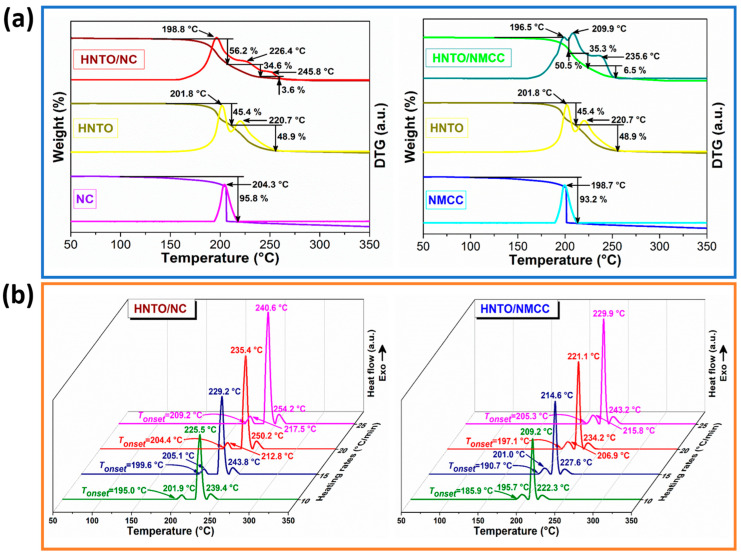
(**a**) TGA/DTG curves of the as-prepared energetic composites and their raw materials at β = 10 °C/min; (**b**) DSC thermograms of the as-prepared energetic composites at different heating rates.

In order to assess the suitability of the designed energetic composites for real-world applications, their thermal stability and decomposition behavior were investigated by TGA and DSC analyses, and the results are illustrated in Figure 4. As can be seen from the TGA/DTG thermograms plotted in Figure 4a, nitrated cellulosic polymers (NC and NMCC) present a single prominent mass loss event (≥95%) at 190–220 °C, whereas HNTO salt undergoes two consecutive decomposition processes at 195–210 °C and 215–235 °C with a weight loss of around 45.4% and 48.9%, respectively. In the case of their chemical mixtures, it is obvious from TGA/DTG curves that both HNTO/NC and HNTO/NMCC composites underwent a three-step of decomposition. The first stage recorded at 180–210 °C with a mass loss of 56.2% for HNTO/NC and 50.5% for HNTO/NMCC is corresponded to the main thermolysis process of nitrated cellulosic chains through thermolytic cleavage of explosophoric O-NO_2_ groups. The two last overlapped decomposition events, which happened in the range of 215–250 °C with a total weight loss of 38.2% for HNTO/NC and 41.8% for HNTO/NMCC, are assigned respectively to the low and high decomposition stages of HNTO, where the detailed mechanism can be found in the work of Yi et al. [27]. Moreover, it is evident from Figure 4a that the composite based on NMCC matrix displays lower onset and major peak decomposition temperatures than that based on pristine NC, which can be explained by the increased nitrogen content and reduced particle size of NMCC with respect to the pristine NC, leading to the acceleration of the thermal degradation [44,45]. Such a statement was also confirmed by Dobrynin et al. [46] and Chen et al. [47], who demonstrated that the replacement of NC with its micro- or nanosized derivatives is very helpful for preparing promising energetic nanocomposites with enhanced thermal reactivity and combustion performance. In addition, the early decomposition of HNTO/NC and HNTO/NMCC composites with respect to their raw nitrated cellulosic matrices can be also promoted by the released reactive species and energy during the decomposition of HNTO.

On the other hand, DSC experiments have been also executed at various heating rates to elucidate the exothermic/endothermic processes that occur and to evaluate the thermo-kinetic parameters that are very important in mastering the thermolysis features of energetic materials. The measured heat flow curves of both elaborated composites at different heating rates (β) are shown in Figure 4b, while the resulting thermal parameters, such as the onset decomposition temperature (*T*_onset_), the maximum decomposition temperature (*T*_peak_), as well as the heat released (∆*H*) at β = 10 °C/min are summarized in Table 2. According to Figure 4b, both energetic composites exhibit three consecutive exothermic processes including the main decomposition peak of the nitrate esters and two other peaks associated with the decomposition of HNTO. These decomposition phenomena are found to be highly dependent on the β, and hence are kinetic events because a faster heating rate reduces the reaction time and delays the decomposition phenomena. Furthermore, it is evident from Table 2 and Figure 4 that the initial and maximum decomposition temperatures of nanostructured HNTO/NMCC composite are lower than those of HNTO/NC one, which corroborate the TGA/DTG findings. This behavior can be attributed to the enhanced interfacial contact between nanostructured nitrocellulose and HNTO, as proved by SEM analysis, which promote the heat and mass transfer within the composite and accelerate its thermal decomposition. This statement can also be justified through the values of the difference between the peak and the onset temperatures (∆*T*) for the different decomposition steps of the HNTO/NMCC composite, which significantly decreased compared to those of the HNTO/NC formulation (Table 2). Another interesting aspect that one can depict is the shift of the exothermic processes of HNTO towards a higher temperature, stipulating that the heat transfer from the reaction zone to the unburned portions of HNTO is facilitated by nitrate esters, which sustains the propagation of the exothermic reaction [48,49]. Indeed, the early decomposition of nitrated cellulosic polymer (NC or NMCC) could be considered as the likely thermolysis trigger of the investigated energetic composites [47,50]. It is interesting to mention based on the above discussion that the thermolysis process of nitrated cellulosic matrices mainly controls the thermal behavior of the elaborated composites. In addition, it is found that the total heat release of HNTO/NMCC (1912.1 J/g) is higher than that of HNTO/NC (1835.8 J/g), confirming once more the effectiveness of using nitrated nanostructured cellulose rather than traditional NC to improve the energetic performance of the designed composite. Besides that, it is important to point out that the new designed HNTO/NC and nanostructured HNTO/NMCC composites have better thermal stability than some reported nitrocellulose-based energetic formulations such as NC/GAP (*T_peak_* = 193 °C, β = 10 °C/min) [18] and NC/HMX (*T_peak_* = 168.2 °C, β = 10 °C/min) [20]. Based on the above thermal results, we can conclude that both nitrated cellulose-rich polymer and HNTO salt may affect the thermolysis of each other via a synergistic effect, and thus such composite can find effective application in the area of solid rocket propellants and explosives.

### 2.5. Determination of the Thermo-Kinetic Parameters

Due to the potential hazardous thermal runway of nitrate ester-based energetic formulations, it is very important to study their thermos-kinetic behaviors to accurately control their reactivity and thermolysis features. Such investigations could not be achieved by simple evaluation of thermal decomposition temperatures obtained from TGA or DSC analyses. Therefore, the obtained non-isothermal DSC data have been further exploited to calculate the key kinetic parameters, namely, the activation energy (*E_a_*), the pre-exponential factor (*Log*(*A*)), and the reaction decomposition model (g(α)). Before discussing each result of the Arrhenius parameters, it seems worthy to define them. *E_a_* is typically defined as the lower energy needed to initiate a reaction for which a higher value means more input energy is required to ensure the ignition of the sample, whereas *Log*(*A*) represents the collision between molecules per time unit, where a higher value implies higher reactant’s reactivity. In most cases, this latter parameter can be effectively determined via a model-free approach using the so-called compensation effect, which stipulates the existence of a linear relationship between *E_a_* and *Log*(*A*) [51,52].

**Figure 5:**
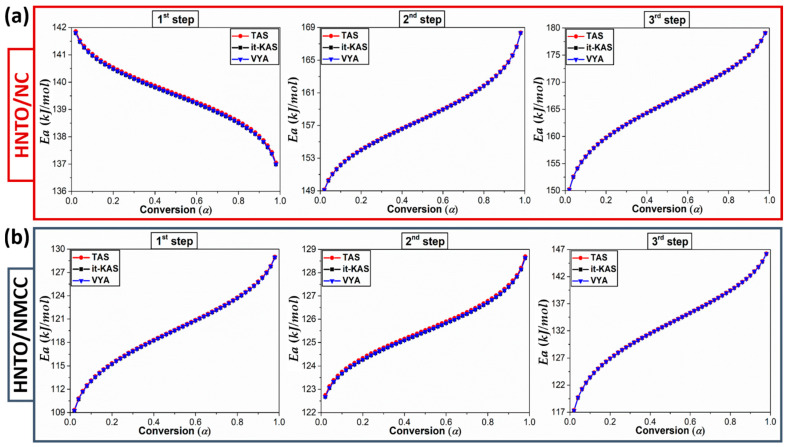
Variation of the activation energy versus the extent of conversion for each decomposition step of (**a**) HNTO/NC composite; (**b**) HNTO/NMCC composite.

**Figure 6:**
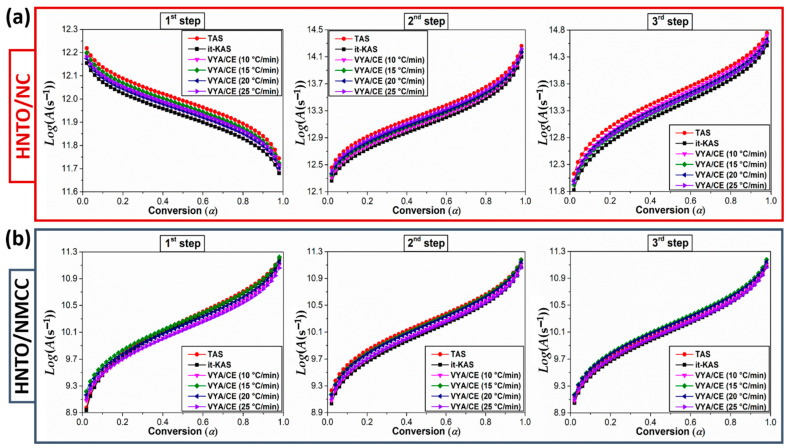
Variation of the decimal logarithm of the pre-exponential factor versus the extent of conversion for each decomposition step of (**a**) HNTO/NC composite; (**b**) HNTO/NMCC composite.

As recommended by the ICTAC, a mathematical deconvolution of the DSC peaks of both energetic composites was performed using the asymmetric Frazer–Suzuki function, which is the most used method to greatly fit the multistep kinetic behaviors. After that, the experimental data were subjected to three isoconversional approaches (TAS, it-KAS and VYA/CE) to compute the kinetic triplet for each decomposition process. The dependence of the Arrhenius parameters with the extent of conversion (*α*) for each thermolysis step that occurred in the designed energetic composites are plotted in Figure 5 and Figure 6, while the average values of *E_a_* and *Log*(*A*) associated with their corresponding confidence intervals, as well as the mathematical reaction mechanisms g(α), are listed in Table 3. The foremost finding is that the calculated Arrhenius parameters using the three isoconversional methods for both energetic composites are in line with each other with a relative deviation lower than 10%, demonstrating the excellent consistency of the performed computations. The high accuracy of the computed Arrhenius parameters using the linear TAS and it-KAS models can be also confirmed by the strong regression coefficient higher than 0.9993. Furthermore, the standard deviation uncertainties introduced in the values of *E_a_* and *Log*(*A*) are within those recommended by the ICTAC (<30%) [53]. Another interesting result is that the evolution profile of *Ea* and *Log*(*A*) vs. the extent of conversion, for each thermolysis stage is similar, which is justified by the energy compensation effects during decomposition [52].

It can be observed from Figure 5 and Figure 6 that the multistep decomposition processes of the as-prepared energetic composites exhibit different trends of Arrhenius parameters as a function of conversion, indicating that their thermokinetic behaviors follow the chemical reaction of the monomolecular energetic materials (NC, NMCC, and HNTO). In the first stage, evaluated at an apparent *E_a_* of 139 kJ/mol for HNTO/NC and 119 kJ/mol for HNTO/NMCC associated with the homolytic cleavage of explosophoric O-NO_2_ groups, HNTO/NC shows a decrease in *E_a_* and *Log*(*A*) values with the extent of conversion, while an opposite trend of these parameters is obtained for HNTO/NMCC. This finding reveals that the beginning of this first decomposition process is much easier in the case of HNTO/NMCC composite, which is expected due to the increased amount of thermally unstable nitrate esters of NMCC, promoting the heat accumulation and hot spot formation within the composite, and consequently elevating its sensitivity against thermal stimuli as outlined by thermal analyses (TGA and DSC). It is worthy to point out that the average value of *E_a_* obtained for the first thermolysis process of HNTO/NMCC is lower than that of HNTO/NC, which are even inferior to those of single NC (172 kJ/mol) and NMCC (156 kJ/mol) matrices [54], indicating that the presence of HNTO significantly improves the process via the enhancement of the heat and mass transfers within the nitrate esters. A similar trend was found in the research work of Chen et al., who reported an increase in the decomposition rate of nitrate esters when an explosive is added [21].

Regarding the second decomposition process, which corresponds to the simultaneous cleavage of nitro, hydrazine, and carbonyl groups accompanied by azole ring breaking, both energetic composites show the same increasing evolution profile of Arrhenius parameters with an apparent dependency on the conversion degree. Such behavior demonstrates that the rate of the second thermolysis process is much easier at the beginning of conversion, which is due to the autocatalytic reaction channels caused by the released radicals and oxidizing species formed during the homolytic splitting of nitrate esters that significantly reduced the initiation energy of pure HNTO molecule broadly ranged between 180–200 kJ/mol [16,43]. This finding suggests that the hot reactive species releases from the decomposition of nitrated cellulosic polymer (NC or NMCC) would show strong catalytic effect only in gas-phase reaction after initial decomposition of HNTO at higher temperature (increased *T_peak_*), resulting in lowering activation energy of decomposition. For the last stage, we can clearly denote from Figure 5 and Figure 6 that both elaborated energetic composites display a growth behavior in Arrhenius parameters versus conversion, providing evidence for the potential catalytic effect of nitrate esters on the gas-phase thermolysis reaction upon initial decomposition of HNTO. In this event, the resultant species from the second decomposition step are adsorbed on the surface of residual HNTO salt, which is then decomposed at a higher temperature into gas-phase products. In addition, the obtained mean values of *E_a_* for the last decomposition stage of HNTO/NC and HNTO/NMCC composites are, respectively, 166 kJ/mol and 133 kJ/mol, which are lower than the common pyrolysis energy range of pure HNTO reported in our previous papers [16,29]. This result highlights once more the extra initiation energy provided by the nitrate ester exothermic decomposition, which certainly leads to the decrease of the activation energy of the decomposition processes. Besides that, it is worthy to highlight that, during the whole decomposition process, the kinetic parameters of the new prepared nanoenergetic composite based on nitrated cellulose nanostructure are found to be lower than that based on the common nitrocellulose, corroborating the thermal findings.

**Figure 7:**
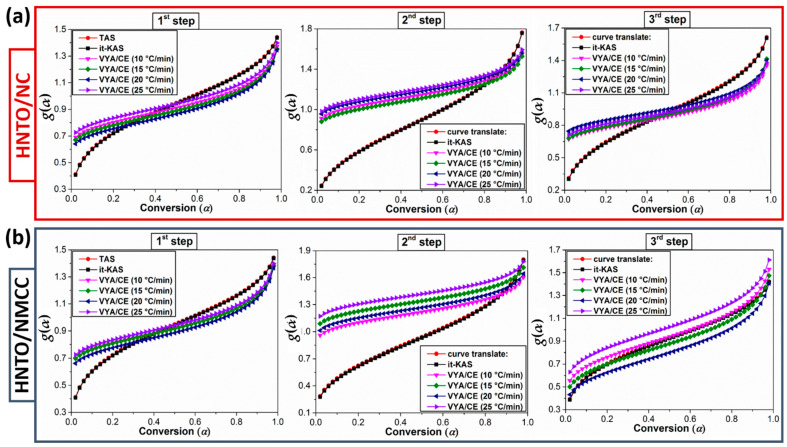
Variation of the integral reaction model versus the extent of conversion for each decomposition step of (**a**) HNTO/NC composite; (**b**) HNTO/NMCC composite.

Another crucial point to consider in the examination of the thermokinetic behavior of the newly developed energetic composites is the assessment of the variation of their most probable reaction models (*g*(*α*)) derived from the employed isoconversional methods. The evolution of the models as a function of conversion is displayed in Figure 7, while the mathematical formula of the models is given in Table 3. It is important to mention that the Vyazovkin non-linear method does not allow obtaining the reaction model, but its combination with the compensation effect can provide numerical values of the experimental *g*(*α*). Among the 41 theoretical models reported in our recent paper [55], the investigated HNTO/NC and HNTO/NMCC formulations follow various reaction mechanisms during their decomposition stages. However, it is found that TAS and it-KAS provide the same integral model for each energetic composite at each thermolysis step. According to these isoconversional kinetic methods, the first decomposition process of both energetic composites is governed by a chemical reaction mechanism (G_1_). In the case of the second and the last decomposition processes, based on the employed linear isoconversional methods, both energetic composites decompose according to a random nucleation mechanism of Avrami-Erofeev (A_5/2_, A_3_, and A_4_). Compared to pure HNTO, the same model categories are obtained and already mentioned in previous work [16,43], indicating that the decomposition process of the nitrate esters does not affect the reaction model unlike the activation energy of the process. Besides that, it is interesting to point out the kinetic results obtained for the thermal behavior of the new developed energetic composites agreed well with the data of the same category of nitrocellulose-based energetic composites found in the open literature [21,56]. Henceforth, the thermokinetic findings of the current work provide new insight into the importance of developing new nanoenergetic composites based on nitrated cellulose nanostructure and HNTO instead of the highly sensitive NG, for promising application in high-performance solid propellants and explosive formulations.

### 2.6. Sensitivity Features

In order to probe the safety performance of the developed energetic composites, their impact and friction sensitivities are tested, and the determined values are displayed in Table 1. It is obvious that the developed energetic composites, comparable to their raw compounds, are insensitive toward friction (FS ≈ 350 N) according to the UN Recommendations on the Transport of Dangerous Goods. Furthermore, their impact sensitivities are found to be relatively similar to that of HNTO explosive but interestingly better than those of nitrated cellulosic polymers, benefiting from the synergetic effect and closer interfacial contact between HNTO and nitrated cellulosic matrices. The uniform dispersion of HNTO in nitrated cellulosic chains would also reduce the formation of the hot spot explosive and hence decrease the sensitivity of the composites. Accordingly, the newly prepared ordinary- and nano-energetic composites present fundamentally acceptable physical stability, rendering them promising candidates for real-world potential use in defense applications.

## 3. Experimental Section

### 3.1. Materials

HNTO with a purity of 99.5% was previously synthesized according to the procedure reported in our recent papers [16,29]. NC and NMCC with a nitrogen content of 12.61% and 13.08%, respectively, were prepared in our laboratory following the same approach mentioned by Tarchoun and coworkers [54].

### 3.2. Theoretical Design of the Composites

**Figure 8:**
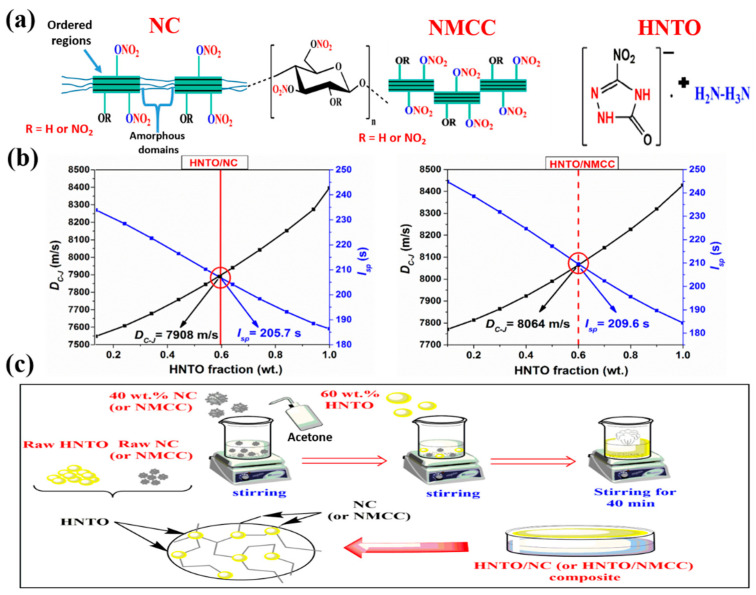
(**a**) Molecular structures of pure NC, NMCC, and HNTO; (**b**) evolution of the specific impulse and detonation velocity of the prepared composites as a function of the HNTO weight percentage; (**c**) preparation procedure of the target energetic formulations.

The thermodynamic calculation is necessary for composites formulation design since their energetic performance could be approximately predicted by theoretical calculation. For this purpose, several thermochemical tools such as EXPLO5 software, ICT thermodynamic code (Institute of Chemical Technology, virgin 2008, Fraunhofer, Germany), and NASA Chemical Equilibrium with Applications (CEA) (NASA, RP-1311, Washington, DC, USA) are widely used. In this study, EXPLO5 thermochemical computer code (OZM Research, Version 6.04, Czech Republic, European Union) was employed to determine the optimal composition of the as-prepared energetic composites, which delivered the optimum values of specific impulse (*I_SP_*) and detonation velocity (*D_C-J_*). The first parameter, which is widely used to compare the efficiency of rocket boosters, is determined by assuming isobaric combustion; whereas the *D_C-J_* and other detonation properties are predicted according to the Chapman-Jouguet (CJ) theory by assuming isochoric combustion [57]. The molecular structures of the pure ingredients are provided in Figure 8a, while the obtained results corresponding to the theoretical specific impulse and detonation velocity are presented in Figure 8b.

### 3.3. Preparation Procedure of the Optimal Composites

HNTO/NC and HNTO/NMCC composites were elaborated from nitrogen-rich mixtures of 60 wt.% HNTO (Energetic Materials Laboratory, Algiers, Algeria) and 40 wt.% nitrated cellulosic matrix (Energetic Materials Laboratory, Algiers, Algeria). It should be noted that the weight percentages of the different ingredients are optimized using the computation method detailed above. As illustrated in Figure 8c, dried NC (or NMCC) was firstly dissolved in a sufficient amount of acetone and stirred at room temperature for 40 min. After that, dried HNTO was added in small portions to the solution under continuous stirring for 30 min. During the mixing process, a few mL of acetone should be added in order to avoid the viscosity decrease of the mixture caused by the high volatility of acetone. Lastly, the resulting composites (HNTO/NC and HNTO/NMCC) were poured into specific aluminum pans, and the residual acetone was eliminated after drying the admixtures in a vacuum oven. It is important to mention that although we experienced no difficulties in handling these energetic materials, production at a small scale with precautionary safety practices (leather gloves and face shield) is strongly encouraged.

### 3.4. Compatibility Assessment

During the steps of preparation and storage of energetic mixtures, incompatibility issues could strongly accelerate the aging process, which results in a change in the thermal stability as well as impairing the safety and operational performance of the formulation. Therefore, testing the compatibility of an energetic material with other compounds is an extremely crucial step in developing new energetic formulations in order to ensure their storage stability and reliability. In this study, the effect of HNTO on the chemical compatibility of nitrated cellulosic polymers has been assessed using DSC standard thermal method. According to the standardization agreement (STANAG) 4147 ed. 2, measurements should be carried out for pure samples and their physical mixtures in a 1:1 mass ratio [28]. In addition, it should be noted that, while STANAG 4147 requirements call for thermal analyses to occur at 2 °C/min, a heating rate of 10 °C/min was taken to minimize time analysis as already reported in the literature [58,59,60]. A series of runs was performed, and an analysis of the data showed no discernable difference in the results. It should be noted that our powder samples (NC, NMCC, and HNTO) were passed through a sieve with a 2 mm opening to obtain effective mixing and a high degree of contact between ingredients during testing. The DSC-based compatibility standard considers that the difference in the maximum peak temperature (Δ*T_p_*) calculated as given in Equation (1) is the main criterion for determining the chemical compatibility.

∆*T_P_* = *T_S_* − *T_M_* (1)

where: *T_S_* and *T_M_* are the exothermic peak temperatures of the single compound and the physical mixture, respectively. The single system is the pure energetic component, whose exothermic peak temperature is the smaller one. According to the guidelines described in STANAG 4147 ed. 2 [28], if ∆*T_P_* ≤ 4 °C, the mixture is determined as compatible; if ∆*T_P_* ≥ 20 °C, the mixture is incompatible; if ∆*T_P_* is between 4 and 20 °C, another method is recommended to evaluate the compatibility.

### 3.5. Characterization Techniques

The morphological structure of raw materials and the homogeneity of their corresponding formulations were analyzed by scanning electron microscopy (SEM) recorded on an FEI Quanta 600 at an accelerating voltage of 5 kV. The samples were coated with 20 nm of carbon to reduce charging effects. Their chemical structure was also characterized by Fourier transform infrared spectroscopy (FTIR) performed using a Perkin Elmer 1600 spectrometer. The spectra were collected in ATR mode in the range of 4000–500 cm^−1^, with an accumulation of 64 scans and a resolution of 4 cm^−1^.

Experimental densities were measured using a Gas pycnometer device, type Accupyc 1340 II electronic densimeter. Ten measurements were performed, and the reported value is the average one. The experimental density values ($\rho_{EXP}$) of the prepared composites were compared to their theoretical maximum densities ($\rho_{TMD}$) using the following formula:

(2) $$\Delta \rho \left(\%\right)=100\times \frac{/\rho_{EXP}-\rho_{TMD}/}{\rho_{TMD}}$$

where Δρ represents the percentage gap between real and theoretical composite density. The theoretical density values of the as-prepared composites were calculated from their respective ingredient densities listed in Table 1.

The thermal decomposition behavior of the involved energetic composites was assessed using thermogravimetric analysis (TGA) and differential scanning calorimetry (DSC) techniques. TGA experiments for about 1–2 mg samples were carried out on Perkin Elmer TG 8000 (mass accuracy 0.1 µg) under a constant nitrogen atmosphere at a heating rate of 10 °C/min from room temperature to 350 °C. DSC measurements were recorded on Perkin-Elmer DSC 8000 analyzer (Perkin-Elmer, Waltham, MA, USA). for around 1 mg samples at different heating rates (10, 15, 20, and 25 °C/min) under an inert atmosphere from 50 °C to 350 °C.

Sensitivities toward impact (IS) and friction (FS) of all samples were determined with a standard BAM drop hammer and friction tester according to STANAG 4489 and STANAG 4487, respectively [61,62]. The limiting values of the impact energy and friction force were determined as the lowest value at which a positive result is obtained from at least one out of six repeated tests.

#### Kinetic Computations

To further elucidate the thermal runaway mechanism of the newly elaborated energetic composites, their thermokinetic behaviors were studied by subjecting the non-isothermal DSC data to isoconversional kinetic analysis. Herein, we followed the recommendation of the International Confederation for Thermal Analysis and Calorimetry (ICTAC), where the reaction rate, at constant conversion (*α*), only depends on temperature (Equation (3) [53]. Basically, the isoconversional approach allows estimating the Arrhenius parameters, namely, the activation energy (*E_a_*) and the preexponential factor (*Log*(*A*)) without requiring, in prior, the reaction model (*g*(*α*)) given by Equation (4).

(3) $$\frac{d\alpha }{dT}=\frac{A_{a}}{\beta }e^{\left(\frac{-E_{a}}{RT}\right)}f\left(\alpha \right)$$

(4) $$g\left(\alpha \right)=\overset{\alpha }{\underset{0}{\int }}\frac{d\alpha }{f\left(\alpha \right)}=\frac{A_{a}}{\beta }\int_{T_{0}}^{T}e^{-E_{a}/RT}dT$$

From DSC curves, the value of *α* is calculated as a ratio of the current heat change ΔH to the total reaction heat $\Delta H{\;}_{total}$:

(5) α=∫t0t(dHdt)dt∫t0tf(dHdt)dt=ΔH ΔH total

Herein, two linear isoconversional models, namely, Trache–Abdelaziz–Siwani (TAS) [55], and the iterative Kissinger–Akahira–Sunose (it-KAS) [63], and one non-linear isoconversional Vyazovkin’s method (VYA) coupled with the compensation effect approach (CE) [52], were computed to determine the hole kinetic triplet (*E_a_*, *Log*(*A*), *g*(*α*)). The calculations were carried out using a local code compiled in MATLAB software.

## 4. Conclusions

In summary, new high-energy dense composites based on HNTO explosive and nitrated cellulosic polymers (NC and NMCC) were successfully elaborated through a casting method. Beforehand, the chemical compatibility of HNTO with NC and NMCC matrices was confirmed by employing DSC standard method. A theoretical performance computation was then conducted to determine the optimal composition of the as-prepared HNTO/NC and nanostructured HNTO/NMCC formulations, which corresponded to 60/40 wt.%, based on the optimum values of specific impulse and detonation velocity using EXPLO5 V6.04 software. Structural characterizations (SEM and FTIR) and density measurements of the designed energetic composites demonstrated their homogeneity with good dispersion of HNTO within the nitrated cellulosic chains, which promoted from NC to NMCC. Compared to the baseline HNTO/NC formulation, the nanostructured HNTO/NMCC composite possessed slightly low decomposition temperatures and activation energies, while a higher heat release is obtained, confirming its improved thermal reactivity. Furthermore, the thermal decomposition of HNTO is also increased when dispersed within the nitrate esters matrix owing to the enhanced heat transfer from the reaction zone to the unburned portions of HNTO, which sustains the propagation of the exothermic reaction.

In addition, the computed isoconversional kinetic approaches showed that the developed energetic composites follow different mechanisms during their decomposition stages, which could change from a chemical reaction (G_1_) to a random nucleation mechanism of Avrami–Erofeev (A_5/2_, A_3_, and A_4_). It was also revealed that both HNTO/NMCC and HNTO/NMCC formulations possessed fundamentally acceptable safety performance with FS and IS around 350 N and 6 J, respectively. In light of these results, it can be concluded that the new developed high-energy dense nanocomposite based on nanostructured nitrocellulose with promising thermal and kinetic features, as well as attractive performance can be considered as a futuristic high-performance formulation for the development of a new generation of composite explosives and solid rocket propellants.

## Figures and Tables

**Table 1:** Densities, impact and friction sensitivities of the investigated samples.

Sample	$\rho_{EXP}\;(g/{\mathrm{cm}}^{3})$	$\rho_{TMD}\;(g/{\mathrm{cm}}^{3})$	Δρ (%)	IS (J)	FS (N)
HNTO	1.820 ± 0.003	/	/	7	360
NC	1.671 ± 0.004	/	/	3	350
NMCC	1.694 ± 0.004	/	/	2	350
HNTO/NC	1.781 ± 0.002	1.760	1.2	6	350
HNTO/NMCC	1.793 ± 0.002	1.770	1.3	6	350

**Table 2:** DSC parameters of the as-prepared energetic composites obtained at β = 10 °C/min.

Sample	1st Decomposition Stage				2nd Decomposition Stage				3rd Decomposition Stage				
	*T*_onset_ (°C)	*T*_peak_ (°C)	∆*T* * (°C)	∆*H* (J/g)	*T*_onset_ (°C)	*T*_peak_ (°C)	∆*T* * (°C)	∆*H* (J/g)	*T*_onset_ (°C)	*T*_peak_ (°C)	∆*T* * (°C)	∆*H* (J/g)	∆*H_T_* (J/g)
HNTO/NC	195.0	201.9	14.2	549.5	218.7	225.5	6.8	950.5	232.7	239.4	6.7	335.8	1835.8
HNTO/NMCC	185.9	195.7	9.8	560.9	204.1	209.2	5.1	994.8	218.8	222.3	3.5	356.4	1912.1

* ∆*T* = *T*_peak_ − *T*_onset_; ∆*H_T_*, total heat release.

**Table 3:** Kinetic triplet for the thermal decomposition of the as-prepared composites.

Sample	Isoconversional Method		*Eα* (kJ/mol)	*Log* (*A(*s^−1^))	*g*(*α*)
HNTO/NC 1st step	TAS		139.49 ± 12.40	11.95 ± 2.84	G_1_ = 1 − (1 − *α)*^2^
	it-KAS		139.54 ± 12.45	11.83 ± 2.83	G_1_ = 1 − (1 − *α)*^2^
	VYA/CE	β = 10 °C/min	139.49 ± 12.35	11.89 ± 1.30	/
		β = 15 °C/min		11.93 ± 1.30	/
		β = 20 °C/min		11.89 ± 1.30	/
		β = 25 °C/min		11.85 ± 1.30	/
HNTO/NC 2nd step	TAS		157.96 ± 9.55	13.25 ± 1.35	A_5/2_ = [*−ln(*1 − *α)*]^2/5^
	it-KAS		157.97 ± 9.55	13.11 ± 1.34	A_5/*2*_ = [*−ln(*1 − *α)*]^2/5^
	VYA/CE	β = 10 °C/min	157.96 ± 9.37	13.15 ± 1.21	/
		β = 15 °C/min		13.18 ± 1.25	/
		β = 20 °C/min		13.20 ± 1.24	/
		β = 25 °C/min		13.22 ± 1.20	/
HNTO/NC 3rd step	TAS		165.93 ± 11.06	13.45 ± 1.86	*A_3_* = [*−ln(*1 − *α)*]^1/3^
	it-KAS		165.96 ± 11.05	13.30 ± 1.85	A_3_ = [*−ln(*1 − *α)*]^1/3^
	VYA/CE	β = 10 °C/min	165.94 ± 8.67	13.41 ± 1.60	/
		β = 15 °C/min		13.32 ± 1.62	/
		β = 20 °C/min		13.38 ± 1.58	/
		β = 25 °C/min		13.35 ± 1.59	/
HNTO/NMCC 1st step	TAS		119.5 ± 7.85	10.27 ± 1.22	G_1_ = 1 −(1 − *α)*]^2^
	it-KAS		119.5 ± 7.90	10.20 ± 1.21	G_1_ = 1 −(1 − *α)*]^2^
	VYA/CE	β = 10 °C/min	119.5 ± 8.70	10.13 ± 0.93	/
		β = 15 °C/min		10.23 ± 0.98	/
		β = 20 °C/min		10.20 ± 0.95	/
		β = 25 °C/min		10.14 ± 0.91	/
HNTO/NMCC 2nd step	TAS		125.4 ± 9.90	10.32 ± 1.80	A_5/2_ = [*−ln(*1 − *α)*]^2/5^
	it-KAS		125.5 ± 9.95	10.20 ± 1.73	A_5/2_ = [*−ln(*1 − *α)*]^2/5^
	VYA/CE	β = 10 °C/min	125.5 ± 10.83	10.24 ± 0.89	/
		β = 15 °C/min		10.30 ± 0.93	/
		β = 20 °C/min		10.27 ± 0.94	/
		β = 25 °C/min		14.24 ± 0.92	/
HNTO/NMCC 3rd step	TAS		133.08 ± 11.15	10.34 ± 1.70	A_4_ = [*−ln(*1 − *α)*]^1/4^
	it-KAS		133.11 ± 11.70	10.22 ± 1.62	A_4_ = [*−ln(*1 − *α)*]^1/4^
	VYA/CE	β = 10 °C/min	133.10 ± 11.65	10.25 ± 1.02	/
		β = 15 °C/min		10.37 ± 1.11	/
		β = 20 °C/min		10.35 ± 1.08	/
		β = 25 °C/min		10.26 ± 1.06	/

## Data Availability

Available data are presented in the manuscript.
